# Storage and Stability of AAV-Containing Fibrin Hydrogels for Retinal Gene Therapy

**DOI:** 10.3390/gels12070591

**Published:** 2026-07-02

**Authors:** Aubrey Berger, Travis Knudsen, Francesca Kopp, David Korda, Mary Lang, Brittni A. Scruggs, Alan D. Marmorstein

**Affiliations:** 1 Biomedical Graduate Program, Mayo Clinic, Rochester, MN 55905, USA; 2 Department of Ophthalmology, Mayo Clinic, Rochester, MN 55905, USAscruggs.brittni@mayo.edu (B.A.S.); 3 Mayo Clinic Alix School of Medicine, Mayo Clinic, Jacksonville, FL 32224, USA; 4 Department of Pediatrics, Mayo Clinic, Rochester, MN 55905, USA

**Keywords:** gene therapy, hydrogel, AAV, retinal disease, storage, stability

## Abstract

Subretinal and intravitreal injection of retinal gene therapy is associated with serious adverse events and poor efficacy. We sought to improve retinal gene therapy delivery by developing fibrin hydrogel encapsulated adeno-associated virus (FE-AAV). Here we investigate conditions for storage and stability for FE-AAV. FE-AAV containing 1.9 × 10^9^ genome copies of AAV2/2-CMV-GFP was manufactured using fibrinogen reconstituted in 0.01 M sodium citrate, pH 7 (NaC) or phosphate-buffered saline containing 0.001% (*v*/*v*) Pluronic F68 (F68). Samples were stored at either −80 °C or 4 °C for up to 16 weeks. Changes in transduction efficiency, and mechanical and physical properties were evaluated. In vitro transduction was significantly (*p* < 0.05) reduced for FE-AAV manufactured with NaC. In contrast, we observed no change in transduction through 16 weeks for FE-AAV made with F68. Physical changes occurred in FE-AAV stored at −80 °C. In contrast to FE-AAV formulated with NaC, FE-AAV formulated with F68 and stored at 4 °C for 16 weeks was essentially equivalent to freshly made FE-AAV and retained the ability to transduce retinal pigment epithelial (RPE) cells in the pig eye. We conclude that FE-AAV formulated with F68 and stored at 4 °C is stable and shows potential for retinal gene therapy for at least 4 months following manufacture.

## 1. Introduction

In 2017, the FDA approved the first in vivo gene therapy, Luxturna^®^ (voretigene neparvovec), a solution of recombinant adeno-associated virus (AAV) for treatment of Leber congenital amaurosis and retinitis pigmentosa due to variants in *RPE65*. Since then, dozens of clinical trials have tested other gene therapies for retinal diseases, many of them AAV-based. Despite approximately 7 additional in vivo AAV gene therapy approvals by the FDA since 2017 for non-ophthalmic indications, there have been no new gene therapies approved for ophthalmic use since Luxturna [1]. Furthermore, of patients receiving Luxturna post-FDA approval, chorioretinal atrophy (CRA) and other vision-threatening severe adverse events were reported in as many as 24.7% of patients [2]. An additional study on patients receiving Luxturna treatment after previously receiving a different viral gene therapy in their contralateral eye (i.e., first eye treated in clinical trial) found that three out of four patients developed CRA [3]. These adverse events are likely attributable to a combination of the subretinal injection method and gene therapy dose. Luxturna and the majority of experimental retinal gene therapies are administered via subretinal injection. This involves inserting a cannula through the neurosensory retina and injecting a volume of fluid containing the gene therapy between the retinal photoreceptors and retinal pigment epithelial (RPE) cells, creating a focal retinal detachment. We and others have reported abnormal retinal changes from subretinal injections in large animal models [4,5,6,7,8,9]. In a systematic review of viral gene therapy clinical trials utilizing subretinal injections, 11.6% (51 of 438) of eyes receiving treatment experienced a severe adverse event [2], the majority of which were retinal degeneration [10,11].

The next most common viral gene therapy delivery method used in clinical trials is intravitreal injection. Intravitreal injections inject the viral solution into the vitreous humor, a viscous gel that fills the posterior cavity of the eye. For intravitreal injection to be effective, the gene therapy must diffuse through the vitreous gel to the retina. Patients receiving viral gene therapy from intravitreal injection exhibit a lower incidence of severe adverse events (3.2%, [2]) than those receiving subretinal injection, most commonly inflammation [2]. However, despite improved safety, only one in 11 intravitreal injection trials met an efficacy endpoint [2]. A third method of delivery of gene therapy to the retina is suprachoroidal injection, which places the gene therapy in the space between the sclera and choroid. According to clinicaltrials.gov, five gene therapy trials (NCT04514653, NCT07313618, NCT05099094, NCT06765980, NCT06458595) are or have employed suprachoroidal injections. To date, no data are published for any of these trials. Some non-human primate results show transduction of the outermost retina layer from suprachoroidal injections, but without penetration into the rest of the retina [12]. Another study in non-human primates suggests that suprachoroidal injections lead to immune response against the transgene, as suprachoroidal injections lead to transgene expression in scleral cells [13]. Thus, challenges associated with vector administration remain a significant barrier to the success of viral gene therapy trials.

To address these issues, our laboratory has developed an AAV-encapsulated solid fibrin-hydrogel (FE-AAV) delivery system as an alternative method of gene therapy administration [4]. Fibrin is formed by the cleavage of fibrinogen by the enzyme thrombin during the blood clotting cascade. Following cleavage, fibrin polymerizes into a fibular crosslinked hydrogel that serves as the matrix of blood clots. Autologous fibrin products have a decades-long history of safe clinical use as tissue glues/sealants [14], including in the eye, where fibrin is the most commonly used bioadhesive [15,16,17,18]. Fibrin is degraded naturally by the body’s anti-clot mechanisms, negating any need for surgical removal or additional materials to induce degradation. This is true even in otherwise avascular areas such as the intra-ocular and subretinal space [4,19,20].

We have sought to take advantage of these properties to improve delivery of viral gene therapy to the retina. In previous work, AAV2-encoding green fluorescent protein (GFP) was added to the gel formulation to create FE-AAV [4]. When adhered to the epiretinal surface of the retina, the fibrin gel degraded within days and the AAV released transversed the neurosensory retina, resulting in transduction of the RPE with fewer adverse events than intravitreal and subretinal injections [4]. With this proof-of-concept study, we have demonstrated a promising technique to improve the delivery of viral gene therapy to the retina; however, those studies used FE-AAV that was manufactured on the day of surgery [4]. For FE-AAV to be deployed clinically, it must be able to undergo release testing following manufacture and maintain stability in inventory prior to shipping, transportation, and on-site storage at the point of care. For this reason, it is critical to determine whether FE-AAV can be manufactured and stored in a way that is stable for an extended period while retaining its ability to be easily degraded in vivo. Storage methods must preserve critical quality attributes (CQAs) of FE-AAV, such as vector potency, structural integrity, overall morphology, degradability, and mechanical strength. In this study, we sought to test the effects of temperature and fibrinogen reconstitution solution on the preservation of FE-AAV properties. Typical storage solutions for AAV include excipients such as Pluronic F68, a surfactant that has been shown to improve thermal stability and preserve AAV capsid integrity [21,22,23,24]. Here, we examine the effect of the reconstitution buffer and temperature on FE-AAV and demonstrate that FE-AAV can be stored for an extended period while retaining potency and other critical properties.

## 2. Results and Discussion

### 2.1. FE-AAV Storage Conditions

Our prior proof of principle study [4] used FE-AAV manufactured the same day as surgery. To guide GMP process development and identify critical process parameters (CPPs) for that workflow, a set of CQAs (Table 1) for FE-AAV was developed. As day-of-use production is impractical in a clinical setting, the CQAs require that FE-AAV can be stably stored with sufficient time to perform release testing and inventory prior to shipping to a potential user. Important CPPs contributing to this CQA include the buffer formulation used for manufacture and storage, and storage conditions, including temperature. FE-AAV used in previous work was formulated with 0.01 M sodium citrate, pH 7.0, (NaC) as the reconstitution solution for fibrinogen. The decision to use this was based on CQAs developed for cell therapy applications [20] and did not take into account the use of AAV in the fibrin gel. In those applications we included aprotinin or tranexamic acid, anti-fibrinolytic compounds that prevent degradation of the hydrogel [4,19,20]. For FE-AAV, we do not want to include anti-fibrinolytics as they result in slower degradation [4,20,25]. Previously, we used NaC to formulate FE-AAV. NaC does not contain any anti-fibrinolytic compound, but the FE-AAV gels were implanted on the day of manufacture.

**Figure 1:**
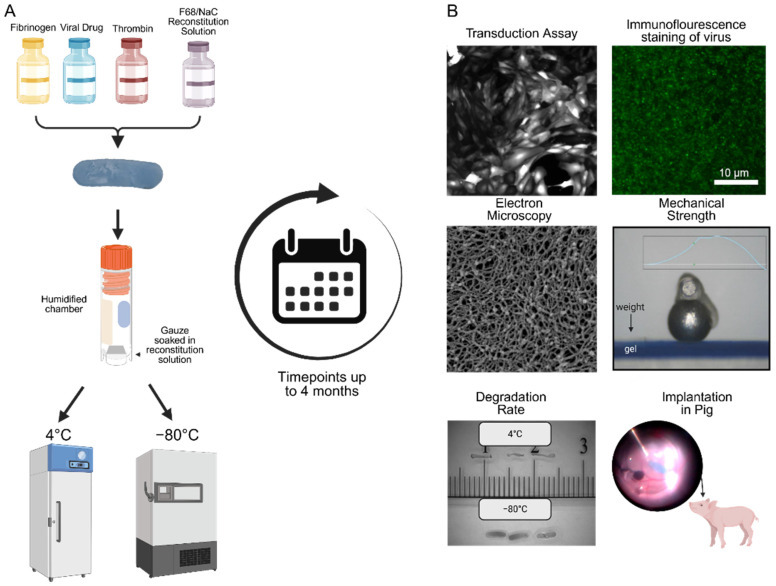
Experimental layout. Overall scheme of storage experiments. (**A**) FE-AAV were made by reconstituting fibrinogen and thrombin in F68 or NaC buffer with GFP-encoding AAV. The FE-AAV were stored in a humidified cryovial at 4 °C or −80 °C. (**B**) FE-AAV characteristics related to CQAs (ST1) were tested using a variety of methods illustrated by representative examples from our results.

In an effort to preserve AAV integrity for storage, NaC was tested against PBS containing 0.001% (*v*/*v*) Pluronic F68 (F68), a buffer known to be more favorable to AAV storage [21]. Trypan blue was added to the F68 and NaC buffers as well because we have previously shown it slows the initial gelation step sufficiently to permit injection molding [4,20,25]. To determine whether we could meet the criteria of storage and stability, FE-AAV were made using NaC or F68 and evaluated to determine which buffer lends more protective effects. FE-AAV designated for storage were adhered to the side of a cryo-vial humidified with the addition of gauze soaked with 100 μL of the corresponding buffer at the bottom (Figure 1A). The FE-AAV were not submerged in a solution (Figure 1A). In a pilot study storage temperatures of −80 °C, −20 °C, 4 °C, and 37 °C were tested. AAV transduction in vitro dropped significantly when stored at −20 °C or 37 °C (Figure A2A) and so these conditions were excluded from further consideration. We next conducted a more rigorous comparison addressing relevant CQAs of NaC vs. F68 stored at −80 °C vs. 4 °C over a 16-week time course (Figure 1B).

**Figure 2:**
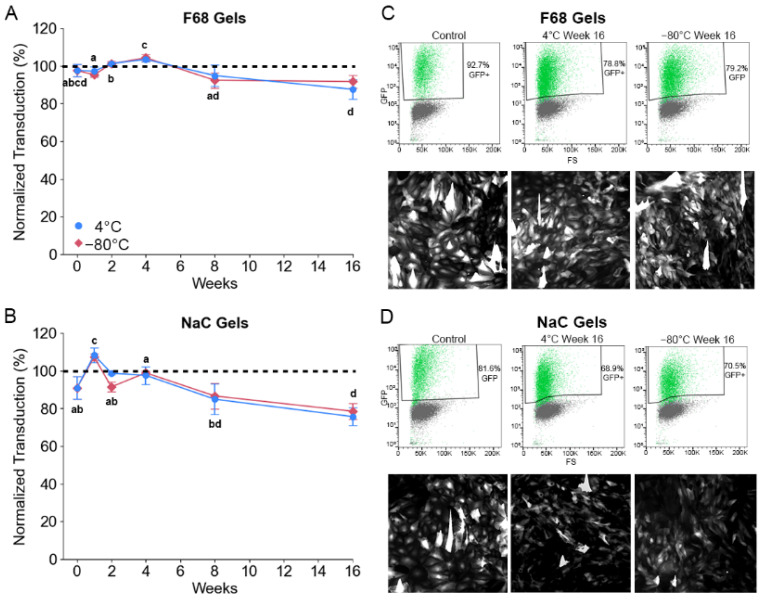
Transduction of stored FE-AAV. Transduction from F68-formulated FE-AAV (**A**) or NaC-formulated FE-AAV (**B**) stored in 4 °C or −80 °C up to four months as assessed by measuring GFP with flow cytometry (average ± sd, *n* = 5 flow cytometry samples). Graphed data are normalized to a positive control. Weeks not sharing lower case letters differ significantly (*p* < 0.05) as determined by the pairwise Wilcoxon test. (**C**) Example of raw flow cytometry results for fresh control F68-formulated FE-AAV and samples stored for four months with an overlay of negative controls in gray. Representative images of GFP expression in live confluent cells are shown underneath. (**D**) Example of raw flow cytometry results for fresh control NaC-formulated FE-AAV and samples stored for four months with an overlay of negative controls in gray. Representative images of GFP expression in live confluent cells are shown underneath.

We chose only to address CQAs (Table 1) that would directly affect storage and stability as outlined in CQA 1: “FE-AAV hydrogels can be stored long enough to accomplish release testing, and shipment to other sites without loss of potency and other properties”. CQA: “FE-AAV hydrogel contains a known dose (vg/µL hydrogel)”, was addressed in Figure 2. CQA 3 was not directly addressed as differences in the ratio of vg/capsids should show as differences in transduction efficiency and the stock AAV used was the same regardless of FE-AAV formulation. To address CQA 4, “FE-AAV can efficiently transduce target cells and express transgene”, transduction assays (Figure 2) and in vitro degradation (Figure 4) assays were conducted. To address CQA 5, “FE-AAV possesses a set of defined mechanical characteristics”, degradation assays (Figure 4), OCT imaging (Figure 5), mechanical testing (Table 2), and electron microscopy (Figure 6) were conducted. Combined, these assays address CQA 1: “FE-AAV hydrogels can be stored long enough to accomplish release testing” (Table 1). Safety tests indicated in CQAs 6 and 7 were not performed since these would not likely be affected by the variables tested.

### 2.2. Viral Transduction from Stored FE-AAV

**Figure A1:**
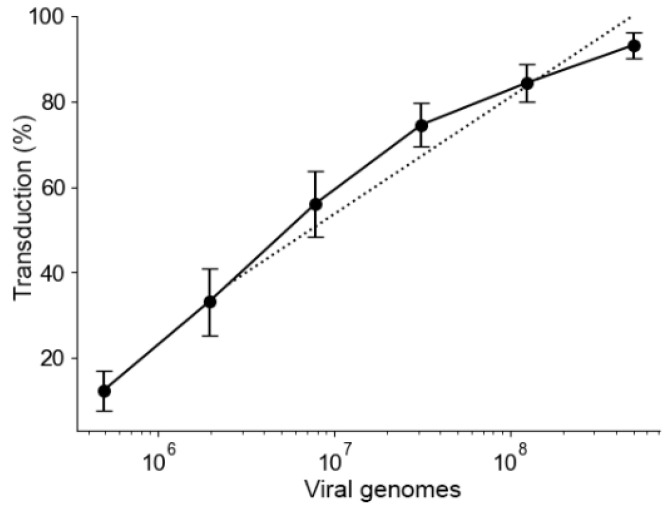
Transduction Standard Curve. Transduction from viral dilutions to create a standard curve (average ± SE, *n* = 4–5 flow cytometry samples). Trendline (dotted): 11.9ln(x) − 137.9 (R = 0.97).

Potency of FE-AAV was tested using an in vitro transduction assay as described previously [4]. Keeping cell plating density and culture conditions constant throughout all assays, a standard curve for the assay was created by performing serial dilutions of AAV solution to determine the relationship between cellular transduction and viral dosage as reported by genome count (gc). It was determined that there is a logarithmic relationship between added gc and the percentage of GFP positive cells (Figure 1A). To determine FE-AAV transduction efficiency, FE-AAV were added to confluent ARPE19 cells and incubated for one week. Transduction efficiency was determined by the percentage of GFP positive cells assessed by flow cytometry (Figure 2A,B). No difference in transduction was observed when comparing control FE-AAV from either formulation. However, during storage, FE-AAV formulated in NaC exhibited a downward trend in in vitro transduction efficiency beginning at week 8 (Figure 2A,B). This became significant (*p* = 0.01) at the 16-week timepoint (Figure 2B) when NaC-formulated FE-AAV, regardless of storage temperature, transduced on average 15% fewer cells than controls. A standard curve was derived from viral dilutions (Figure A1). From this standard curve, a logarithmic relationship between viral dosage and percent transduction was found (Figure A1).

This suggests a 68% (±43%) reduction in viable AAV at the 16-week timepoint for FE-AAV formulated using NaC. While a slight downward trend in transduction efficiency was noted for F68 beginning at week 8, no significant (*p* < 0.05) loss of transduction was observed in the F68-formulated FE-AAV at either temperature throughout the 16-week study.

**Figure 3:**
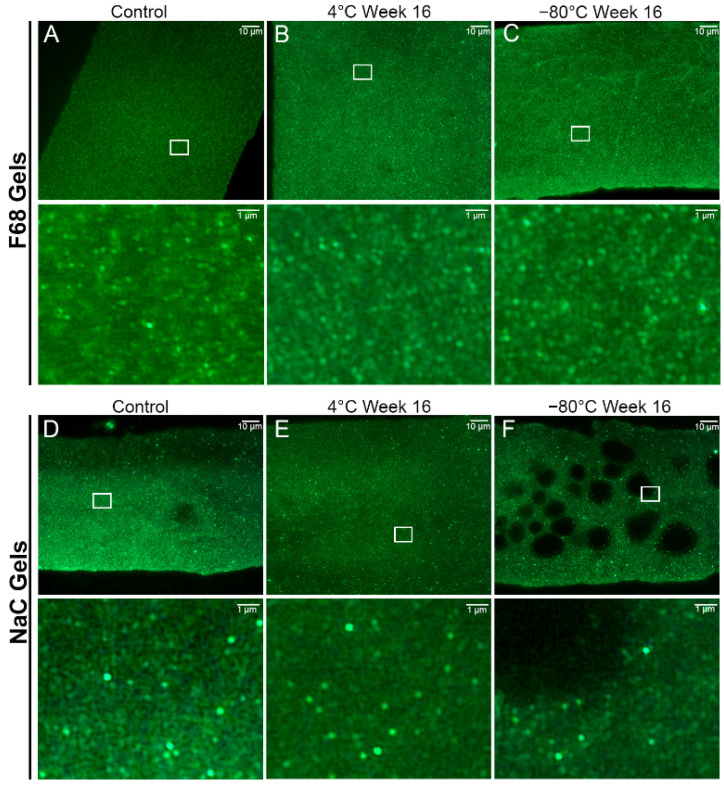
Viral distribution in stored FE-AAV. Immunofluorescent staining of AAV (*n* = 1 gel). (**A**) Control F68-formulated FE-AAV compared to those stored for 16 weeks at 4 °C (**B**) and −80 °C (**C**). Magnified version of white box showed underneath. (**D**) Control NaC-formulated FE-AAV compared to those stored for 16 weeks at 4 °C (**E**) and −80 °C (**F**). Magnified version of the area in the white boxes are shown underneath.

**Figure 4:**
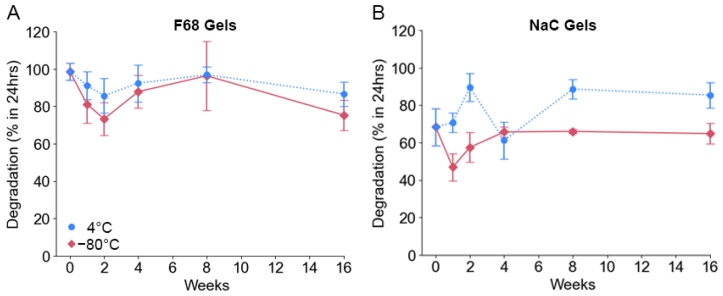
In vitro degradation of FE-AAV. Percent degradation (average ± sd, *n* = 3 gels) of F68-formulated FE-AAV (**A**) and NaC-formulated FE-AAV (**B**) after 24 h of digestion with plasmin at 37 °C.

Due to the loss of transduction from NaC-formulated FE-AAV that was stored for 16 weeks, we performed immunofluorescence staining of FE-AAV for AAV capsids (Figure 3). AAV particles appeared uniformly distributed in F68-formulated FE-AAV in control and the 16-week timepoints (Figure 3A–C) regardless of storage temperature. In contrast, NaC-formulated FE-AAV exhibited an apparent reduction in AAV particles (Figure 3D–F) at 16 weeks. Furthermore, in NaC-formulated FE-AAV stored at −80 °C, large holes appeared in the gel interior (Figure 3F) without any apparent increase in the density of AAV particles in the remaining gel matrix (Figure 4).

The most critical property of FE-AAV is potency, as measured by the ability to transduce target cells to express the transgene carried by the AAV vector. As the virus used in our validation study targets the RPE, we used the ARPE19 cell line to model RPE. When adapting FE-AAV to target different cell types, different cell lines will need to be used. Here, the in vitro transduction efficiency assay established a saturable logarithmic dose-potency relationship. Storage temperature did not affect FE-AAV transduction, but notable differences were observed when comparing NaC and F68. Previous research has shown that the addition of the surfactant Pluronic F68 to solutions of different AAV serotypes improves viral stability and infectivity [21,23]. Our data reinforce those findings, as no change in transduction was detected in F68-formulated FE-AAV. In contrast, NaC-formulated FE-AAV exhibited a reduction in transduction efficiency that began trending downward following 8 weeks of storage regardless of temperature. That change was statistically significant (*p* = 0.01) by 16 weeks and was the equivalent of a 68% (±45%) reduction in viable AAV as measured by gc. Immunofluorescence staining of AAV capsids suggested that NaC-FE-AAV had a lower density of observable AAV particles compared to control gels at both 4 °C and −80 °C and gels stored at −80 °C exhibited large holes that were not present in controls or gels stored at 4 °C. Since the gels were not submersed in a storage buffer, the lower density of AAV particles cannot be the result of diffusion. In contrast, the distribution of AAV capsids appeared unaltered when comparing F68-FE-AAV controls to gels stored for 16 weeks regardless of storage temperature.

### 2.3. In Vitro Degradation

**Figure A2:**
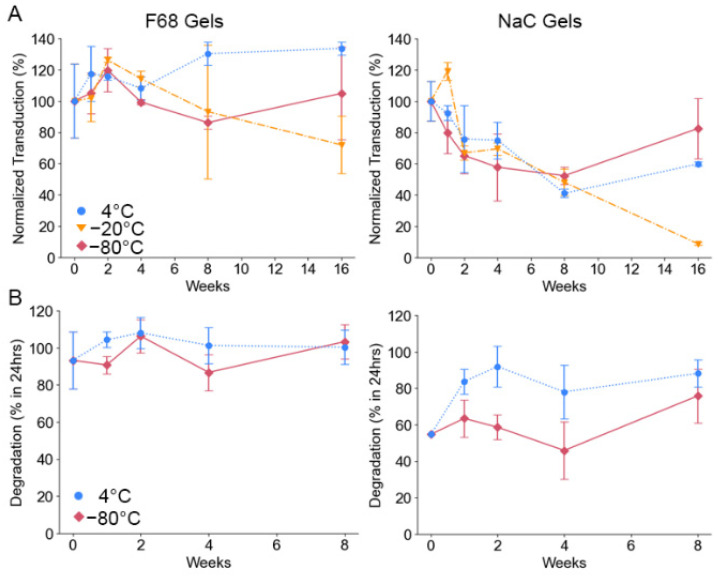
(**A**) Pilot Study Transduction Assay. Transduction from F68-formulated FE-AAV or NaC-formulated FE-AAV stored in 4 °C, −20 °C, or −80 °C up to four months as assessed by measuring GFP with flow cytometry (average ± sd, *n* = 3 wells). (**B**) Percent degradation (average ± sd, *n* = 3 gels, control of NaC *n* = 1, control of F68 *n* = 2 gels) of F68-formulated FE-AAV and NaC-formulated FE-AAV after 24 h of digestion with plasmin at 37 °C.

Degradation in vivo is a critical property of FE-AAV and permits full release of AAV from the fibrin gel [4]. To test whether degradation was altered by storage or formulation, we performed an in vitro gel degradation assay. FE-AAV gels were degraded by the fibrinolytic enzyme plasmin, the principal enzyme responsible for in vivo fibrinolysis [26], for 24 h. Degradation was quantified by assay of dissolved peptides as previously described [4]. When comparing the control groups for NaC (68.3 ± 9.8%, *n* = 3) vs. F68 (98.6 ± 4.5%, *n* = 3), the NaC control degraded 31% less than the F68 control (*p* < 0.01) which degraded almost completely. There was a trend of NaC gels stored at 4 °C degrading more than NaC controls, but only week 2 reached statistical significance (*p* = 0.045). If we exclude week 4 (which was unchanged relative to controls and degraded less than other stored 4 °C NaC gels) as an outlier, collectively NaC gels stored at 4 °C degraded more than controls (*p* = 0.028). Otherwise, there would be no difference between degradation of 4 °C NaC gels and controls. The phenomenon of NaC stored at 4 °C degrading more than controls was also observed during our preliminary study (Figure A2B). The incomplete degradation of fresh NaC gels after 24 h is also comparable to previous work [4]. NaC gels stored at −80 °C did not exhibit any changes in degradation compared to controls. For F68 FE-AAV gels there appeared to be a slight trend towards less degradation following storage, but as a group, F68 FE-AAV gels stored at 4 °C or −80 °C did not differ significantly from controls.

Degradation of FE-AAV is an important CQA. Release of AAV from FE-AAV is partially dependent on degradation of the fibrin gel [4], and failure to degrade could pose a potential safety risk to the patient that could require further intervention. F68-formulated FE-AAV degraded more easily than NaC-formulated FE-AAV and was not significantly altered by storage time or temperature.

### 2.4. FE-AAV Gel Morphology

**Figure 5:**
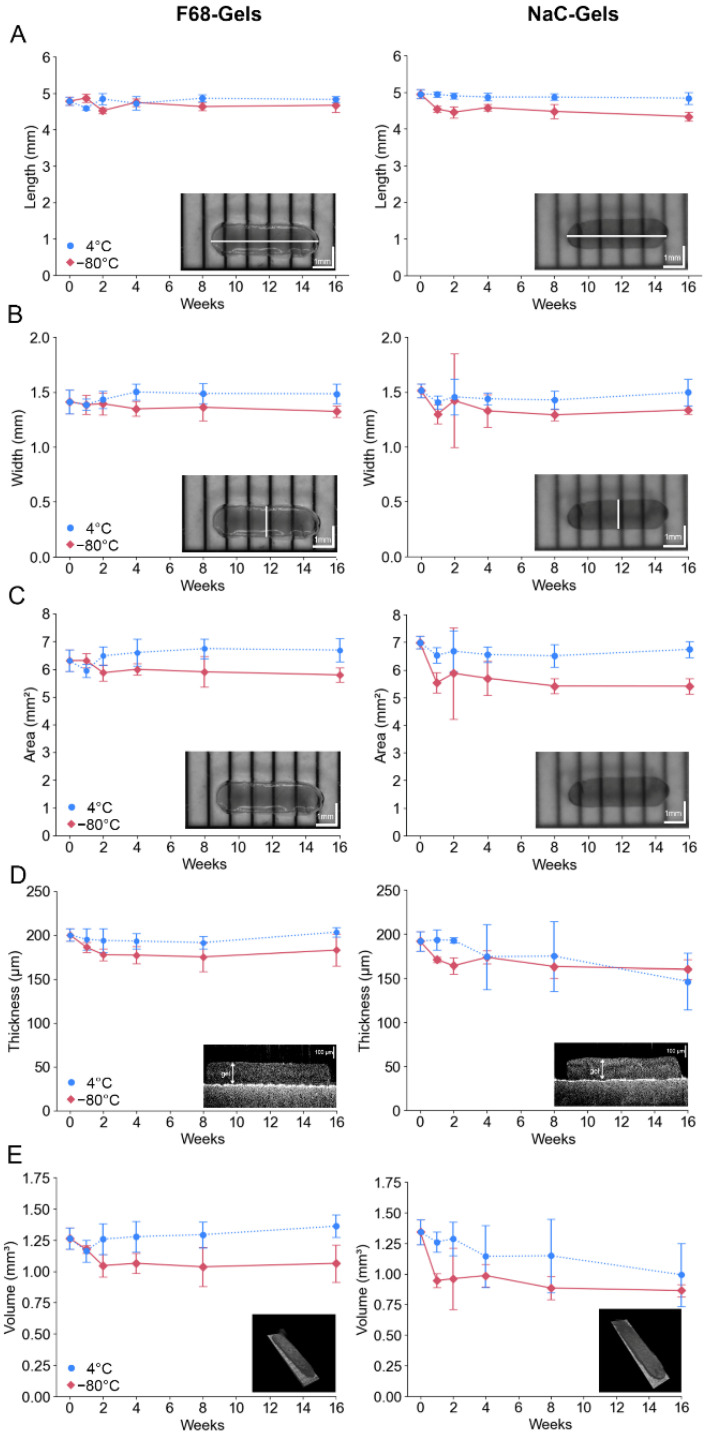
Morphology of FE-AAV gel across 16 weeks. (**A**) Length of gel as measured in direction shown in inset, for F68- and NaC-formulated FE-AAV, respectively (*n* = 5 gels). (**B**) Width of gel as measured in direction shown in inset for F68- and NaC-formulated FE-AAV, respectively (*n* = 5 gels). (**C**) Area of gel as calculated for F68- and NaC-formulated FE-AAV, respectively (*n* = 5 gels). (**D**) Thickness of gel as measured by OCT as shown in inset, for F68- and NaC-formulated FE-AAV, respectively (*n* = 5 gels). (**E**) Volume of gel as calculated for F68- and NaC-formulated FE-AAV, respectively (*n* = 5 gels). Inset picture shows 3D reconstruction from sequential OCT scans. Data are average ± sd.

At each timepoint, the length (Figure 5A), width (Figure 5B), and thickness of FE-AAV were measured (Figure 5D). Area (Figure 5C) and volume (Figure 5E) were then calculated from those measurements. After one week of storage at −80 °C, NaC gels were reduced in length, and this reduction was sustained until week 16. Individual timepoints were not significantly different from control, but taken as a group, NaC gels stored at −80 °C were on average 9.5% shorter than controls (*p* < 0.001). NaC gels stored at 4 °C and F68 gels stored at 4 °C or −80 °C did not exhibit any measurable change in length. Despite some abnormal variability in NaC gels stored at −80 °C for 2 weeks, neither temperature nor formulation caused a significant difference in gel width compared to controls. NaC gels stored at −80 °C were on average 13.3% thinner than controls (*p* < 0.04). The NaC-formulated gels stored in 4 °C additionally show a large amount of variation in thickness (Figure 4B). This was caused by a subset of gels in later timepoints being unusually thin, a phenomenon that did not occur in gels formulated with F68. F68 gels were reduced in thickness after one week of storage at −80 °C, and this reduction was sustained until week 16. Individual timepoints were not significantly different from control, but taken as a group, F68 gels stored at −80 °C were on average 10.2% thinner than controls (*p* < 0.001). No difference was observed for F68 gels stored at 4 °C. Despite no significant differences in volume between individual timepoints and controls, the average calculated volume of all F68 gels stored at −80 °C was 14.8% smaller than F68 control gels (*p* < 0.004) and for all NaC gels stored at −80 °C average calculated volume was 30.9% smaller than NaC control gels (*p* < 0.001). No reduction in volume was observed for F68 gels stored at 4 °C. A non-significant trend of reduction in volume was observed for NaC gels stored at 4 °C.

Both F68- and NaC-formulated FE-AAV shrank after storage at −80 °C, and both exhibited holes. The shrinkage is probably the result of dehydration associated with freezing [27]. Shrinkage was greater in NaC gels, which lost 30.9% of their volume vs. 14.8% for F68 gels. When stored at 4 °C, NaC-formulated FE-AAV showed large variability in volume after time in storage. Many gels exhibited severe thinning, something we did not observe at early timepoints for NaC or F68 at any timepoint. One explanation for this could be stretching of the gel. Stretching of fibrin clots causes a reduction in volume as a result of changes in the molecular structure and expulsion of water [28]. However, it is unlikely the thinning of 4 °C NaC-formulated FE-AAV at later timepoints can be attributed to unintentional stretching, as there was no severe thinning in other groups of the same batch, and no elongation of gels was observed. Overall, the −80 °C storage condition causes all FE-AAV gels to shrink in volume, while 4 °C preserves FE-AAV characteristics in F68-formulated AAV gels.

### 2.5. Stiffness

The stiffness of FE-AAV gels was tested by calculating the Young’s modulus with a microindenter as previously described [4]. No difference was observed from our previously reported Young’s modulus of ~40–50 KPa [4,19], nor were there any differences observed as a function of storage temperature or formulation (Table 2). We did however note that physical handling of gels stored at −80 °C for either formulation was more difficult than handling gels stored at 4 °C, as −80 °C stored gels were flimsier and less adhesive to surfaces.

**Table 2:** Young’s modulus (KPa) of FE-AAV gels.

	F68 4 °C	F68 −80 °C	NaC 4 °C	NaC −80 °C
Week 0 (Control)	21 ± 11 (5)		40.7 ± 25 (4)	
Week 1	44.7 ± 31.6 (5)	36.1 ± 17.6 (5)	29.2 ± 9.5 (5)	42.7 ± 35.8 (5)
Week 2	50.6 ± 10.2 (2)	40.2 ± 11.9 (5)	40.7 ± 18.9 (4)	56.3 ± 9 (4)
Week 4	55.9 ± 18.3 (5)	50.7 ± 29.1 (3)	80.4 ± 17.9 (4)	57.8 ± 9.3 (4)
Week 8	30.9 ± 29.4 (7)	23 ± 15.6 (5)	38.1 ± 21 (6)	42.7 ± 18.7 (6)
Week 16	43.1 ± 33.9 (5)	50 ± 4.6 (5)	61.6 ± 65.5 (3)	61 ± 29.5 (5)

Data are average ± sd (*n* gels).

Changes in mechanical features during storage and handling could underlie the subtle changes observed in degradation efficiency of the fibrin matrix, affect the ease of handling during surgical implantation, and impact the propensity of the gel to become damaged by handling or to damage retinal tissue when implanted. Since dosing is dependent on the gel volume it is important that the FE-AAV gel retain sufficient resiliency to be handled by an operator loading it into a surgical inserter, storage device, or by a surgeon placing the FE-AAV gel, without increasing stiffness to a level that could damage the retina when placed.

### 2.6. Hydrogel Microarchitecture

**Figure 6:**
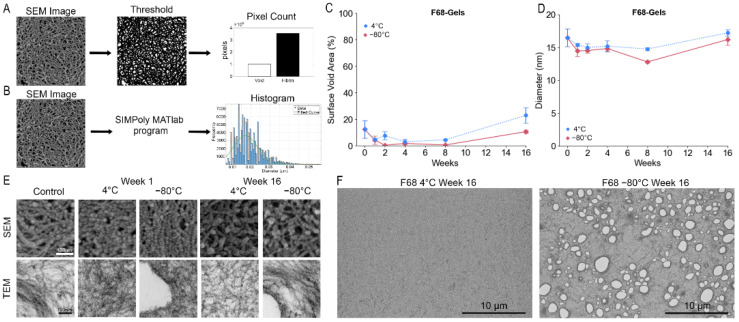
Microarchitecture of stored FE-AAV. Microarchitecture of F68-formulated FE-AAV across 16 weeks. (**A**) Graphical description of surface void measurement process. SEM images are thresholded to distinguish fiber from background; the area of background and fiber is calculated by measuring number of black and white pixels. (**B**) Graphical description of fibril diameter measurement process. SEM images are processed in SIMPoly: A Matlab-Based Image Analysis Tool [29]. The program returns a histogram of all fibril diameters along with the average, number of fibers measured, and standard deviation. (**C**) Changes in surface void area (average ± sd, *n* = 1 gel, *n* =3 technical replicates) as a function of temperature and time in storage. (**D**) Changes in fibril diameter (average ± se, *n* = 1 gel, *n* = 3 technical replicates) as a function of temperature and time in storage. (**E**) SEM and TEM images of FE-AAV at control, week 1, and week 16 timepoints respectively. (**F**) 3D rendering of serial block-face SEM images of FE-AAV stored for 16 weeks at 4 °C or −80 °C. Note the holes of ≤2 µm in the gel stored at −80 °C and absence of those holes in the gel stored at 4 °C.

NaC-formulated FE-AAV exhibited a reduced transduction efficiency in vitro after 16 weeks of storage at either 4 °C or −80 °C (Figure 1). Following storage at −80 °C, holes in excess of 10 µm were common within NaC-formulated FE-AAV gels and could be easily seen by light microscopy (Figure 3F). For these reasons, only F68-formulated FE-AAV were considered for further study. The F68-formulated FE-AAV gel fibril structure stored at either temperature differed from control gels. Void area at the surface of the gel was determined by analysis of scanning electron microscopy (SEM) images (Figure 6A). However, FE-AAV stored at either temperature for 16 weeks exhibited an increase in void area (Figure 6C). FE-AAV stored at 4 °C had a higher void area at 16 weeks than all other timepoints (*p* < 0.05); however, collectively, FE-AAV stored at 4 °C did not differ statistically from the control. FE-AAV stored at −80 °C had a higher void area at 16 weeks than weeks 8, 4, and 2 (*p* < 0.05). Collectively, FE-AAV stored at −80 °C exhibited an average decrease in void area of 72% compared to controls (*p* < 0.001), and a decrease of 60% compared to gels stored at 4 °C (*p* < 0.001). To try to understand the change in surface void volume, we used an open-source MATlab program [29] to quantify fibril diameters in the SEM images (Figure 6B). At both temperatures, the fibril diameters were unchanged relative to control (Figure 6D). This suggests that the reduction in volume observed in gels stored at −80 °C (Figure 5E) is due to contraction rather than fibril loss. Despite observable differences in microarchitecture for all stored gels, this property does not directly impact a CQA because it does not confer any risk to the patient. Therefore, these results should be considered exploratory and this assay will likely not be included in future product quality testing.

Using transmission electron microscopy (TEM), holes were observed in the interior of F68 FE-AAV gels stored at −80 °C (Figure 6E,F). Unlike NaC-formulated FE-AAV, these holes were typically ≤2 µm in diameter and generally too small to be observed easily by light microscopy (Figure 3C). A 3D image compiled from serial block-face SEM scans reveals that the gels are composed of groups of densely packed fiber bundles which extend in all directions (Figure 6F, Video S1). The holes observed by TEM are apparent in the 3D reconstructed image for F68 FE-AAV stored at −80 °C, but those holes are absent from the gels stored at 4 °C (Figure 6F, Video S1, Video S2) which were indistinguishable from controls.

Surfactants can have cryoprotective effects on frozen protein samples, as surfactants prevent adsorption of proteins to ice crystals [30]. The cryoprotectant effect of F68 may explain why the holes observed in NaC gels which were >10 µm in diameter were so much larger than the holes observed in F68 gels which were <2 µm. Though not formally quantified, we noticed that FE-AAV gels stored at −80 °C were flimsier and more difficult to handle than gels of either formulation stored at 4 °C. F68 gels exhibited substantially smaller diameter holes than NaC suggesting that a more comprehensive future evaluation of cryoprotectants and cryoprotectant concentrations could lead us to a formulation that can be stored at −80 °C while retaining acceptable physical characteristics.

### 2.7. In Vivo Transduction

**Figure 7:**
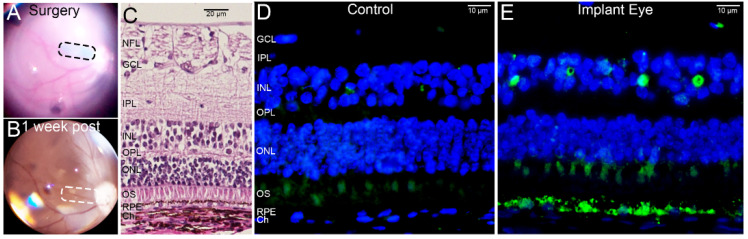
In vivo validation of stored FE-AAV. Fundus image of FE-AAV adhered to the epiretinal surface taken during surgery (**A**) shows a blue oval implant adjacent to the optic nerve head and sitting above a retinal artery. Note the pattern of blood vessels for comparison with a fundus image from the same eye one week later (**B**) showing that the FE-AAV implant has degraded. The area where the implant was placed is indicated by the white dashed line. Hematoxylin and eosin (H&E) staining (**C**) shows normal retinal architecture in an eye receiving a stored FE-AAV implant. Immunofluorescent staining for GFP (green in (**D**,**E**)) shows no staining in the non-implanted control eye (**D**). Nuclei (blue) in (**D**,**E**) are stained with DAPI. Ch = choroid, RPE = retinal pigment epithelium, OS = outer segments, ONL = outer nuclear layer, OPL = outer plexiform layer, INL = inner nuclear layer, IPL = inner plexiform layer, GCL = ganglion cell layer, NFL = nerve fiber layer.

**Figure A3:**
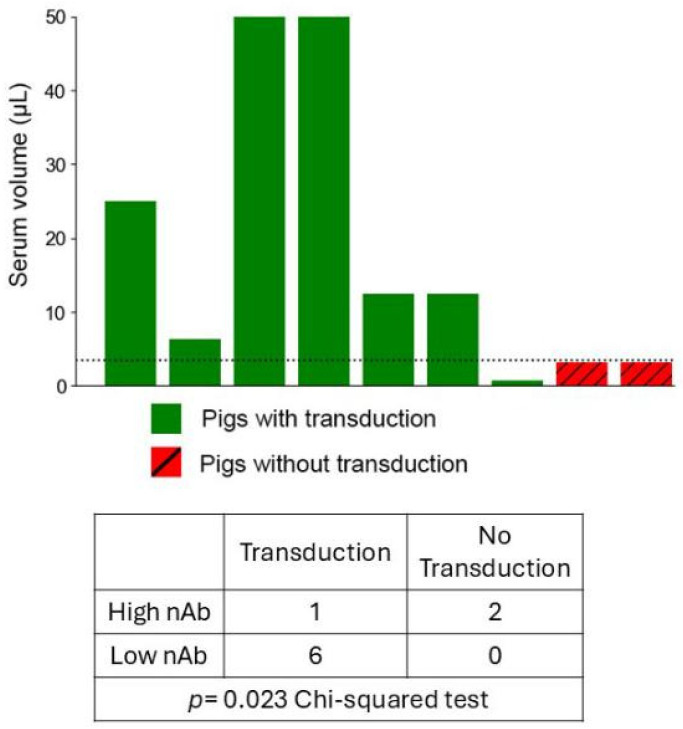
Neutralizing antibody assay. Pre-op serum was collected from pigs and plated by serial dilution across ARPE19 cells dosed with 2.5E8gc virus. The volume of serum that quenched 95% of GFP signal (measured by plate reader after week of incubation) was recorded. Samples that required 3.5 μL or less serum to quench GFP were considered to have high neutralizing antibody (High nAb), and samples that required more serum were considered low neutralizing antibody (Low nAb). When comparing pigs with observable transduction FE-AAV implant (various doses, sample surgical procedures) to their neutralizing antibody levels, High nAb we significantly more likely to a have no transduction.

FE-AAV formulated with F68 and stored at 4 °C retained their in vitro transduction efficiency, degradation properties, physical characteristics, elasticity, and microarchitecture, and were otherwise indistinguishable from freshly manufactured gels. We next tested whether F68-formulated FE-AAV stored at 4 °C for 16 weeks retained the ability to transduce RPE cells in vivo using domestic pigs. FE-AAV formulated using F68 and stored for 16 weeks at 4 °C were surgically adhered to the epiretinal surface of three female juvenile pigs as demonstrated previously [4] (Figure 7A). FE-AAV gels were degraded in the eye within 1 week (Figure 7B), just as previously observed [4]. After 4 weeks, pigs were sacrificed and eyes processed for paraffin histology. Sections were stained with H&E (Figure 7C) or for GFP by immunofluorescence (Figure 7D,E). Retinal anatomy was normal in all eyes receiving FE-AAV (Figure 7C). OCT and fundus examinations conducted at 1-week, 2-week, and 4-week timepoints revealed eyes were normal. One pig that was determined to have a high pre-operative titer of AAV neutralizing antibodies (1 μL neutralized 1.6 × 10^8^ gc) did not express the GFP reporter strongly. Since we used >500 μL of autologous serum applied directly to the FE-AAV gel to attach it to the retina, we concluded that failure to express GFP in this pig was due to neutralization of AAV. This has been observed in other pig studies previously (Figure A3). Moving forward, we will use purified thrombin in place of autologous serum to adhere the FE-AAV gel to the retina. While the untreated contralateral eyes of all pigs were also negative for GFP expression (Figure 7D), GFP expression was observed in the RPE of eyes receiving FE-AAV from the remaining two pigs (Figure 7E). This transduction pattern is similar to transduction observed from freshly made NaC gels [4]. These data provide substantive in vivo support that FE-AAV formulated with F68 and stored at 4 °C for 16 weeks retains the ability to transduce RPE cells in vivo.

## 3. Conclusions

This study demonstrates that FE-AAV constructed as described can be stored long term while maintaining select CQAs and it serves as a basis for storage protocol development for future versions of FE-AAV during GMP process development. The data show that formulation using F68 is superior to NaC (the approach that we have used in the past [4,20]), and that 4 °C is superior to −80 °C in terms of storage and stability. Future studies will seek to further optimize the FE-AAV formulation to permit longer term storage. Further modification could include changes in the type and concentration of surfactant(s) and cryoprotectants to permit storage of frozen gels while retaining mechanical properties and the development of optimized storage containers for use in 4 °C or frozen storage. For example, sugars such as trehalose and sucrose have been used to preserve AAV integrity in formulations for lyophilization [31,32]. Glycerol has also been used as a stabilizer for frozen AAV preparations [23,33]. Those studies will rely on the novel assays reported herein to characterize FE-AAV and will likely include a more comprehensive analysis of dose and potency including genome count and viral particle count. Since the presence of <2 µm holes in F68-formulated FE-AAV stored at −80 °C did not affect potency, future studies will likely assess CQAs based on mechanical properties of the FE-AAV gel in preference to ultrastructural ones. These would include, in addition to indentation, testing of tensile strength, compression, and bending. As FE-AAV is adapted to treat specific indications, different constructions and payloads of virus will be employed, and potency assays will have to adjusted as appropriate. On the basis of the data generated, we conclude that FE-AAV formulated using F68 can be stored for at least 16 weeks at 4 °C without loss of potency or other examined CQAs and that there is a strong potential to identify formulations and conditions permitting even longer storage times.

## 4. Materials and Methods

### 4.1. FE-AAV Hydrogel Preparation

All steps in production of fibrin hydrogels were performed aseptically. Fibrin hydrogels were produced using 4 mL Tisseel™ tissue glue kits (Baxter, Deerfield, IL, USA; NDC#00338-4302-04) with the following modifications. Fibrinogen was resuspended in either 0.01M sodium citrate, pH 7.0, containing 0.12% (*w*/*v*) trypan blue (NaC) or 5.6 mM Na_2_HPO_4_, 1.06 mM KH_2_PO_4_, 154 mM NaCl, pH 7.4, 0.12% (*w*/*v*) trypan blue, and 0.001% (*v*/*v*) Pluronic F68 (F68). The subsequent preparation of the gel containing AAV2/2-CMV-GFP (AAV2[SSAAV.CMV.EGFP.WPRE.SV40pA], Packgene, Houston, TX, USA) added to the thrombin solution was performed as previously described [4]. Gels were incubated at 37 °C for 3 h to cure. Molds were opened in a biosafety cabinet and cured gel blanks were punched using a sterile custom stainless steel oval punch. Each blank yielded approximately 60–80 ovals of 1.5 × 5.1 × 0.2 mm containing 1.9 × 10^9^ gc of AAV2/2-CMV-GFP. Within one hour, gels were packed in cryovials by adhering to the side wall of the vial. Each vial contained a 1 cm^2^ piece of sterile gauze soaked with 100 μL of their respective formulation buffers. Prepared in a biological safety cabinet, the gauze was compressed at the bottom of the vial to create a humidified chamber without contacting the gel (Figure 1A). FE-AAV that were frozen were placed in a pre-cooled Mr. Frosty™ Freezing Container (Thermo Scientific, Waltham, MA, USA, Cat#5100-0001). All −80 °C samples were thawed directly before characterization.

### 4.2. Physical Characteristics

Immediately after FE-AAV preparation, and at each timepoint, gel thickness was measured using a Spectral-Domain Optical Coherence Tomography imaging system (Lumedica OQ Labscope, Durham, NC, USA) as before [4,20]. FE-AAV were measured while in PBS in order to prevent dehydration. For thickness measurements, each gel was measured at least five times across its length and those measurements were averaged. Immediately afterward, each FE-AAV gel was imaged using a Leica stereomicroscope and its length and width was measured using ImageJ (version 1.54p) calibrated to a ruler photographed along with the sample (Figure 5A). To account for the oval shape of the gel, area was calculated according to the following equation:

Area = rectangle + circle = Width ∗ (Length − Width) + π ∗ (Width/2)^2^ (1)

Volume was calculated from thickness and area.

### 4.3. Electron Microscopy and Analysis

At each timepoint, FE-AAVs were fixed in Trump’s fixative and delivered to the Mayo Clinic Microscopy and Cell Analysis Core for scanning electron microscopy (SEM) and transmission electron microscopy (TEM) as previously described [4,19,20]. Three representative images were taken per gel. All images used for analysis were taken at 50,000× magnification. Surface void analysis was completed in MATLAB R2024b. Images were binarized, and the void was measured as the percent of pixels in the background compared to the foreground. The threshold value was selected by using the histogram tool in ImageJ to measure the pixel values of the background; the highest pixel value that removed the background was selected for the threshold (Supplementary Materials S3). Fibril average diameter and standard deviation was analyzed using the Matlab SIMPoly tool [29].

Samples for serial block-face scanning electron microscopy (SBFSEM) were prepared using an Osmium-Thiocarbohydrazide-Osmium (OTO) protocol previously described [34]. Sectioning and imaging of sample was performed using a VolumeScope 2 SEM™ (Thermo Fisher Scientific, Waltham, MA, USA). Imaging was performed under a low vacuum mode to suppress charging artifact, with a starting energy of 3.0 keV and beam current of 0.10 nA. The final magnification was 13,490×. Serial sectioning of 50 nm thickness allowed for imaging at 5 nm × 5 nm × 50 nm spatial resolution. Stacks of 260 images were acquired per region of interest. Image analysis was performed using Amira™ Software, 2020.3 (Thermo Fisher, Waltham, MA, USA). Image stacks were registered using the rigid alignment module and aligned images were used to create a flipbook video and volume renderings.

### 4.4. Histology

Fibrin-AAV gels were fixed overnight in Trump’s fixative, then in neutral buffered formalin for 24 h, and processed into paraffin. They were sectioned into 5 μm and left out overnight for dehydration before staining. Sections were deparaffinized by two successive incubations in xylene for 3 min each, followed by rehydration in absolute ethanol for 1 min and 95% ethanol for 1 min, then blocked for 1 h in 2% BSA (*w*/*v*) with 0.3M glycine in PBS (5.6 mM Na_2_HPO_4_, 1.06 mM KH_2_PO_4_, 154 mM NaCl, pH 7.4) at room temperature. Sections were stained for AAV with a rabbit anti-AAV polyclonal antibody (Invitrogen, PA5-22815) diluted 1:250 in 2% BSA with 0.3 M glycine in PBS for 1 h at room temperature, washed and then reacted with goat anti-rabbit IgG conjugated to Alexa flour 488 (Invitrogen, Waltham, MA, USA, cat# A11008) diluted 1:500 in PBS for 2 h at room temperature. Slides were then washed in PBS, treated with Vector^®^ Autofluorescence Quenching Kit (Vector Labs, Newark, CA, USA, Cat# SP-8400-15) according to the manufacturer’s instructions for five minutes, washed in PBS, and mounted in Fluoromount-G.

Processing of pig eyes for paraffin histology was performed as described previously [4,25] following fixation in Davidson’s fixative. For GFP immunofluorescence staining, sections were deparaffinized in the same manner as the FE-AAV and subjected to heat-induced antigen retrieval using an Aptum Biologics 2100 Antigen Retriever with a basic EDTA buffer (Electron Microscopy Sciences, Morgantown, PA, USA). Sections were first incubated with 3% hydrogen peroxide solution for 1 h (as part of Alexa Fluor™ 488 Tyramide SuperBoost kit (Invitrogen ref# B40912)). They were then blocked in the SuperBoost kit’s 1× blocking buffer for 1 h. Subsequently they were stained for GFP using mouse anti-GFP monoclonal antibody 4B10B2 (Novus) diluted 1:500 in TBST (20 mM Tris, 150 mM NaCl, Tween^®^ 20 detergent 1% (*w*/*v*)) + 5% BSA (*w*/*v*) overnight. Following washing with PBS, sections were incubated with the SuperBoost kit’s secondary antibody for 1 h. According to the manufacturer’s instructions, sections were incubated with the SuperBoost Tyramide reagent for 10 min. Following washing with PBS, nuclei were stained with DAPI and slides were mounted with Fluoromount-G.

### 4.5. Degradation Assay

Stock solutions of plasminogen (R&D Systems) at 200 µg/mL and tissue plasminogen activator (tPA) (Sigma-Aldrich, Burlington, MA, USA) at 100 µg/mL were prepared in sterile water for injection (Gibco, Paisley, Scotland, UK). Plasminogen was diluted to a working concentration of 25 µg/mL using sterile water for injection and activated via the addition of 1/12th volume of the tPA stock solution. A total of 50 µL of activated plasmin solution was added to an oval-shaped gel of 1.5 × 5.1 × 0.22 mm and incubated at 37 °C for 24 h. At the endpoint, any remaining gel was removed from the solution and photographed, and the solution stored at −20 °C prior to assay for total protein using a BCA protein assay kit (Abcam, Cambridge, UK) as before [4].

### 4.6. Biomechanical Strength Testing

Mechanical testing was conducted using a spherical indenter (radius = 0.25 mm) on a MicroTester G2 (CellScale, Waterloo, ON, USA) to determine the Young’s modulus in the Mayo Clinic Biomechanics Core Facility as described previously [4].

### 4.7. Transduction Assay

All transduction assays were performed using ARPE19 cells plated at 25,000 cells per well in a 96-well plate. Cells were fed three times per week with DMEM/F12 supplemented with 10% (*v*/*v*) fetal bovine serum and a 1% (*v*/*v*) antibiotic solution containing penicillin, streptomycin, and amphotericin B (Gibco, Cat# 15240062). Cells were maintained in a humidified incubator at 37 °C in a 95% air/5% CO_2_ atmosphere with either FE-AAV or AAV2/2-CMV-GFP, or no AAV for one week. After one week, TrypLE (Gibco) was used to detach cells from plate. Wells were combined in pairs resulting in six replicates for each condition assayed, then washed once with DMEM/F12 and twice with PBS before staining with Ghost Dye (Cytek ref# 13-0865-T100, 1:1000) for 30 min at 4 °C. Cells were subsequently washed twice with PBS before resuspension in FACS buffer (PBS containing 5% (*v*/*v*) FBS) and protected from light until analysis by flow cytometry to determine the percentage of GFP-expressing cells as described previously [4]. All data were normalized to the percentage of GFP-expressing cells receiving AAV2/2-CMV-GFP at 2.5 × 10^8^ gc per well. Positive controls used the same lot of AAV used in manufacture of FE-AAV and were performed on the same 96-well plate as experimental samples. Using the Levene’s test, the transduction dataset was determined to have non-homogeneous variances, so the non-parametric Kruskal–Wallis and pairwise Wilcoxon tests were used to determine significance.

### 4.8. FE-AAV Implantation in Pigs

All animal procedures were approved by the Institutional Animal Care and Use Committee of Mayo Clinic and conducted in accordance with the Association for Research in Vision and Ophthalmology Statement for the Use of Animals in Ophthalmic and Vision Research. Three 2-month old female pigs underwent surgery to implant FE-AAV containing 1.9 × 10^9^ gc of AAV2/2-CMV-GFP formulated using F68 and stored for 16 weeks. Surgery was performed as described previously [4], on the right eye, leaving the left as a control. On the day of the surgery, autologous blood was collected in BD Vacutainer^®^ SST^™^ tubes. After 30 min at room temperature, blood was centrifuged at 1700× *g* for 10 min and serum collected. The serum was stored at room temperature for less than 30 min before use. To adhere the gel, this autologous serum (>500 μL) was injected around the gel to promote adherence to the retina. Pigs underwent post-operative OCT and fundus exams at 1 and 2 weeks and were euthanized 4 weeks after surgery as described previously [4].

### 4.9. Statistics

Measurements between temperature groups and timepoints were tested with a one-way or two-way ANOVA followed by Tukey–Kramer or Wilcoxon test unless otherwise stated. Comparisons across groups greater than three used the Bonferroni correction. Equal variance between groups was determined using Levene’s test. All analyses were performed in R for windows version 4.5.1, with the exception of power analysis which was done with G*Power version 3.1.9.7. Graphs were created with Python’s Matplotlib 3.10.0.

## 5. Patents

B.A.S. and A.D.M. are inventors on a patent application related to this work filed by Mayo Clinic (U.S. Patent Application no. US-2025/017789). A.D.M. is an inventor on US Patent nos. 11,679,180 and 12303617, “Methods and Materials for using fibrin supports for retinal pigment epithelium transplantation”, held by the Mayo Clinic. A.D.M is an inventor on patent applications related to this work filed by the Mayo Clinic (US Patent Application nos. US-2023/0414826-A1 and US-2022/0000664-A1).

## Figures and Tables

**Table 1:** CQAs for FE-AAV.

CQA	Risk to Patient	CPPs	Assay(s)
1. FE-AAV hydrogels can be stored long enough to accomplish release testing, and shipment to other sites without loss of potency and other properties.	Inconsistent dosing, loss of potency resulting in no therapeutic effect, difficulty handling during surgery.	• Buffer composition • Storage temperature • Storage humidity • Storage packaging	• Visual inspection • OCT imaging (Figure 5) • In vitro cell transduction & expression (Figure 2) • In vitro degradation (Figure 4) • AAV diffusion • Deformation test (Table 2) • TEM (Figure 6) • SEM (Figure 6)
2. FE-AAV hydrogel contains a known dose (vg/µL hydrogel).	Inconsistent dosing affects potency and could cause SAE’s leading to inflammation, CRA, loss of vision, or death.	• Formulation • Gelation	• Visual inspection • OCT imaging (Figure 5) • vg content (PCR)
3. FE-AAV hydrogel contains a known ratio of vg/capsids.	Too many empty capsids can diminish potency and cause SAE’s potentially including inflammation, CRA, loss of vision, or death. Under-dosing risks ineffective therapy.	• Temperature during manufacture • Temperature during storage • Buffer formulation • Maintenance of sterility during manufacture and storage	• Total capsid/volume • Ratio empty vs. full capsid
4. FE-AAV can efficiently transduce target cells and express transgene.	Degradation of vector during production/storage can cause failure to transduce target cells diminishing potency.	• Temperature during storage • Buffer formulation • Maintenance of sterility during manufacture and storage	• In vitro cell transduction & expression (Figure 2) • In vitro degradation (Figure 4) • AAV diffusion
5. FE-AAV possesses a set of defined mechanical characteristics.	• Inconsistent dosing limiting potency • Surgical failure to deploy resulting in traumatic injury or treatment failure • Poor placement or failure to adhere to retina causing diminished potency, inflammation, or traumatic injury • Failure to degrade diminishing potency and potentially obscuring vision.	• Punching • Packaging • Temperature during storage • Buffer formulation • Failure to degrade resulting in diminished potency, inflammation, loss of vision	• Visual inspection • OCT imaging (Figure 5) • In vitro degradation (Figure 4) • Deformation test (Table 2) • Tensile strength (Table 2) • 3-point flexural test • TEM (Figure 6) • SEM (Figure 6)
6. FE-AAV is free of microbial contaminants.	Sepsis, infection, endophthalmitis, death	• Manufacturing under sterile/aseptic conditions • Addition of excipients to enhance sterility and reduce infection/inflammation • Packaging	• Sterility USP<71> • Mycoplasma USP<63> • Endotoxin USP<85>
7. FE-AAV-Hydrogels are free of potentially toxic manufacturing residuals.	Cytotoxicity, sensitization, hemocompatibility, pyrogenicity, genotoxicity, carcinogenicity, reproductive, and developmental toxicity.	Use biocompatible materials in manufacture of all materials that come in contact with FE-AAV	ISO 10,993 biocompatibility testing of molds, storage, and surgical devices.

## Data Availability

The original data presented in the study are openly available from the authors upon request.

## Supplementary Materials

The following supporting information can be downloaded at https://www.mdpi.com/article/10.3390/gels12070591/s1.

## Abbreviations

The following abbreviations are used in this manuscript:

FE-AAV	Fibrin-Encapsulated Adeno-Associated Virus
AAV	Adeno-Associated Virus
F68	Phosphate-Buffered Saline with 0.001% Pluronic F68
NaC	Sodium Citrate
PBS	Phosphate-Buffered Saline
CRA	Chorioretinal Atrophy
CQA	Critical Quality Attribute
GMP	Good Manufacturing Practice
gc	Genome Count
GFP	Green Fluorescent Protein
H&E	Hematoxylin and Eosin
DAPI	4′,6-diamidino-2-phenylindole
RPE	Retinal Pigment Epithelium
Ch	Choroid
OS	Outer Segments
ONL	Outer Nuclear Layer
OPL	Outer Plexiform Layer
INL	Inner Nuclear Layer
IPL	Inner Plexiform Layer
GCL	Ganglion Cell Layer
NFL	Nerve Fiber Layer

## Appendix A

To calculate viral genomes from trendline:

$${Viralge\;nomes=107,821\;*\;e}^{0.084*Transduction}$$

To calculate error of viral genomes:

$${{Error}_{viral\;genomes}=9057\;*\;e}^{0.084*Transduction}*\sigma_{Transduction}$$

To calculate error of percent difference in viral genomes:

$${Error}_{\%}=\sqrt{(\frac{{Error}_{fresh\;gel}}{{Average}_{fresh\;gel}}{)}^{2}+(\frac{{Error}_{stored\;gel}}{{Average}_{stored\;gel}}{)}^{2}+\%}$$
